# Small RNA sequencing reveals metastasis-related microRNAs in lung adenocarcinoma

**DOI:** 10.18632/oncotarget.15968

**Published:** 2017-03-07

**Authors:** Iben Daugaard, Morten T. Venø, Yan Yan, Tina E. Kjeldsen, Philippe Lamy, Henrik Hager, Jørgen Kjems, Lise Lotte Hansen

**Affiliations:** ^1^ Department of Biomedicine, Aarhus University, DK-8000 Aarhus C, Denmark; ^2^ Department of Molecular Biology and Genetics, Interdisciplinary Nanoscience Center (iNANO), Aarhus University, DK-8000 Aarhus C, Denmark; ^3^ Department of Pathology, Aarhus University Hospital, DK-8000 Aarhus C, Denmark; ^4^ Department of Molecular Medicine, Aarhus University Hospital, DK-8200 Aarhus N, Denmark; ^5^ Department of Clinical Pathology, Vejle Hospital, DK-7100 Vejle, Denmark

**Keywords:** miRNA-seq, lung adenocarcinoma, metastasis, FFPE, microRNA

## Abstract

The majority of lung cancer deaths are caused by metastatic disease. MicroRNAs (miRNAs) are posttranscriptional regulators of gene expression and miRNA dysregulation can contribute to metastatic progression. Here, small RNA sequencing was used to profile the miRNA and piwi-interacting RNA (piRNA) transcriptomes in relation to lung cancer metastasis. RNA-seq was performed using RNA extracted from formalin-fixed paraffin embedded (FFPE) lung adenocarcinomas (LAC) and brain metastases from 8 patients, and LACs from 8 patients without detectable metastatic disease. Impact on miRNA and piRNA transcriptomes was subtle with 9 miRNAs and 8 piRNAs demonstrating differential expression between metastasizing and non-metastasizing LACs. For piRNAs, decreased expression of piR-57125 was the most significantly associated with distant metastasis. Validation by RT-qPCR in a LAC cohort comprising 52 patients confirmed that decreased expression of miR-30a-3p and increased expression of miR-210-3p were significantly associated with the presence of distant metastases. miR-210-3p tumor cell specificity was evaluated by *in situ* hybridization and its biomarker potential was confirmed by ROC curve analysis (AUC = 0.839). Lastly, agreement between miRNA-seq and RT-qPCR for FFPE-derived RNA was evaluated and a high level of concordance was determined. In conclusion, this study has identified and validated metastasis-related miRNAs in LAC.

## INTRODUCTION

Lung cancer has the highest mortality rate of all human cancers and is every year liable for approximately 1.5 million deaths worldwide [1, 2]. Lung cancer is a heterogeneous disease with two primary subtypes, small cell lung cancer (SCLC) and non-small cell lung cancer (NSCLC). About 85% of all newly diagnosed lung cancers are NSCLCs and they are further subdivided into adenocarcinomas, squamous cell and large cell carcinomas. Lung adenocarcinoma (LAC) is the most common NSCLC subtype and accounts for approximately 40% of all lung cancer cases [3]. The overall 5-year survival rate of lung cancer is 15%, but it varies extensively depending on how advanced the cancer is at the time of diagnosis [4, 5]. Patients diagnosed with localized lung tumors currently have a 50% chance of surviving at least 5 years following their diagnosis, but this number drops to 25% if the disease has spread to regional lymph nodes and to merely 5% for patients diagnosed with distant metastatic lesions [3]. It is therefore not the primary lung tumor itself, but rather the metastatic spread of the disease that can be held accountable for lung cancers continuously high mortality rate. This is the current situation for most human malignancies as 90% of all cancer deaths are caused by metastatic disease [6, 7]. It is therefore critical to elucidate the molecular mechanisms that characterize the metastatic process.

MicroRNAs (miRNAs) are a class of small non-coding RNAs (∼22nt in length) that regulate gene expression through repression of target messenger RNA (mRNA) expression [8]. More than 50% of all protein-coding genes are currently believed to be under strict microRNA regulation and alterations in miRNA expression can therefore have severe consequences and contribute to the pathogenesis of human malignancies [8–11].

Aberrant miRNA expression has also been implicated in the development and metastatic progression of lung cancer [12–14]. In 2004, Takamizawa *et al*. published one of the first reports of miRNA dysregulation in lung cancer, demonstrating that reduced expression of let-7 was associated with decreased postoperative survival. The role of let-7 in lung cancer has since been extensively investigated and it has been shown to exercise its tumor suppressive function by targeting multiple critical oncogenes, including *MYC* and *RAS* [15–17]. Numerous other oncomiRs and tumor suppressor miRNAs have been identified in NSCLC and in 2013, Vosa *et al*. published a comprehensive meta-analysis identifying a miRNA meta-signature of seven upregulated (miR-21, miR-210, miR-182, miR-31, miR-200b, miR-205, miR-183) and eight downregulated (miR-126-3p, miR-126-5p, miR-30a, miR-30d, miR-486-5p, miR-451a, miR-143, miR-145) miRNAs in NSCLC [18–20]. Furthermore, increased expression of miR-135b was recently identified as an independent predictor of poor overall survival in NSCLC and shown to promote lung cancer metastasis *in vitro* and *in vivo* by targeting multiple genes in the Hippo pathway, including *LATS2*, *β-TrCP* and *NDR* [21]. Similarly, miR-193a has been identified as an inhibitor of NSCLC metastasis by downregulating the ERBB4/PIK3R3/mTOR/S6K2 signaling pathway and miR-29b has been shown to suppress proliferation, migration and invasion abilities of lung cancer cells by targeting *PTEN* and *MMP2*, as well as being significantly associated with lymph node metastasis in NSCLC [22, 23].

P-element-induced wimpy testis (PIWI)-interacting RNAs (piRNAs) are another type of small non-coding RNAs (26-33nt) and with approximately 20.000 genes in the human genome, piRNAs parallel protein coding genes and vastly exceed miRNAs in abundance (∼2.000 genes) [24, 25]. The role of piRNA in human disease has not been studied as extensively as miRNA, but they have recently been recognized as co-players in the development and progression of human cancers, including lung cancer [26, 27]. In fact, piR-55490 was recently reported to be downregulated in lung cancer patient specimens and to be significantly associated with poor overall survival. Moreover, piR-55490 was shown to function as a potent tumor suppressor by inducing degradation of mTOR mRNA in a miRNA-like manner and thereby suppressing the activation of the Akt/mTOR pathway, leading to inhibition of lung cancer cell proliferation [28].

RT-qPCR and hybridization-based microarray platforms have previously been the methods of choice to study aberrant miRNA expression in lung cancer, but these technologies will only allow expression analyses of a limited number of known miRNAs. With the advent of next-generation sequencing (NGS), a powerful new tool for global miRNA expression analysis has emerged (miRNA-seq). A couple of studies have successfully employed miRNA-seq to profile differences in miRNA expression between primary lung tumors and tumor-adjacent normal lung, as well as to study the expression of circulating miRNA in serum and peripheral blood from NSCLC [29–32]. However, miRNA-seq has, to the best of our knowledge, not previously been employed to study the miRNA transcriptome in relation to lung cancer metastasis. In this study, we aimed to characterize the miRNA transcriptome in non-metastasizing and metastasizing LACs with paired brain metastases using miRNA-Seq, as well as to validate our findings in two LAC metastasis cohorts by RT-qPCR.

## RESULTS

### miRNA-seq

**Figure 1:**
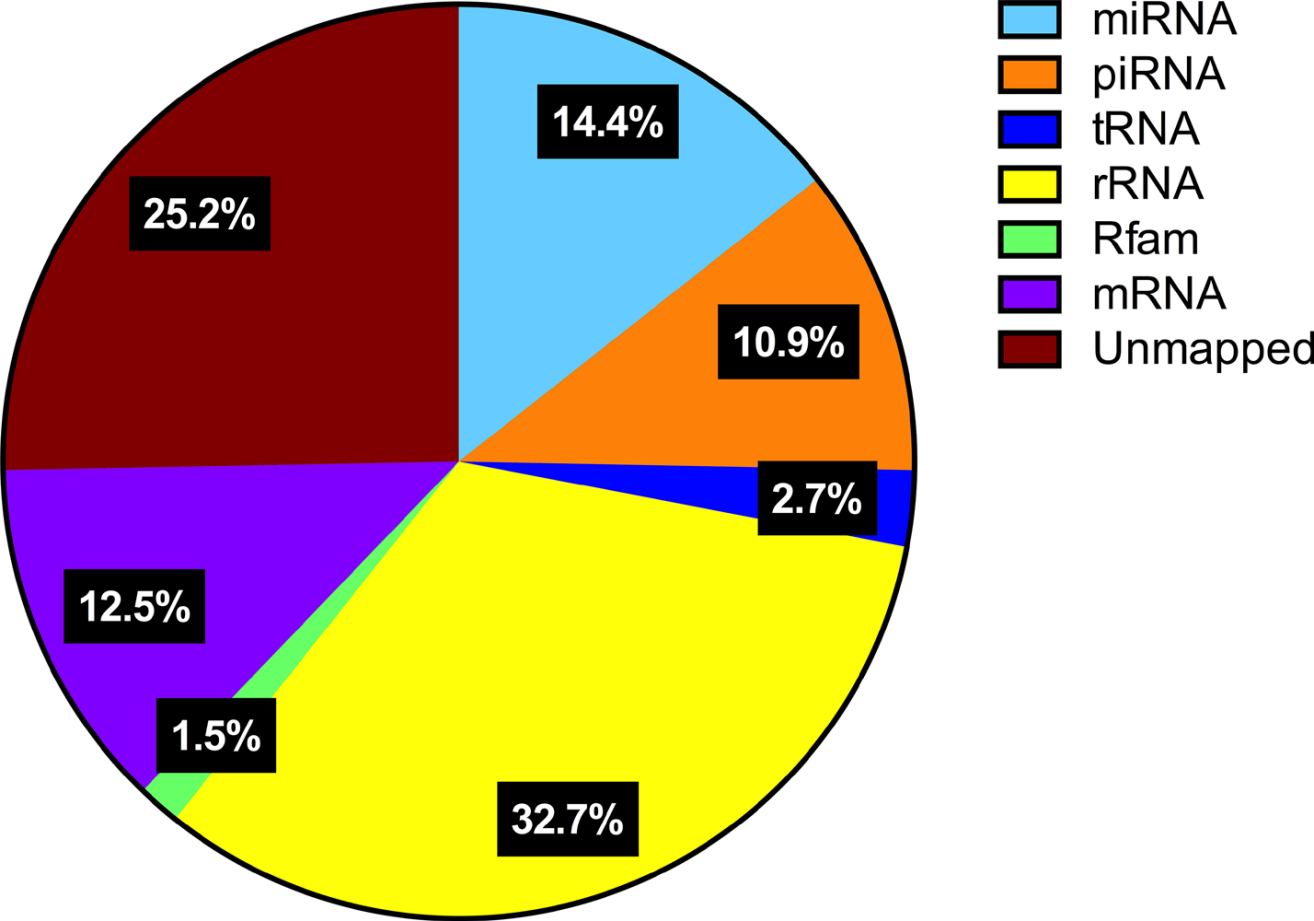
RNA species detected by miRNA-seq Pie chart demonstrating the mean distribution of different RNA species detected by miRNA-seq in all sequenced samples.

In order to characterize the miRNA transcriptome in relation to lung cancer metastasis, we selected primary LACs from 8 patients without detectable distant metastatic lesions and a minimum of 5 years of recurrence-free survival, as well as primary LACs and paired brain metastases from 8 patients that suffered from distant metastatic disease at the time of diagnosis to undergo miRNA-seq analysis. The 16 patients were paired on gender, age, smoking status, T-stage and the relative proportion of tumor cells in the surgical resections. The clinical characteristics of the selected patients are shown in Supplementary Table 1. The RNA integrity in the 24 selected patient samples (8 LACs without metastases, 8 LACs with metastases and 8 paired brain metastases) was assessed prior to preparing the small RNA libraries (Supplementary Figure 1A). All samples demonstrated a high degree of RNA degradation as observed by the predominant presence of small RNA fragments (< 500 bp) and the determined RIN values, which ranged from 2.20 to 2.50. Such low RNA quality is, however, what can be expected for RNA extracted from formalin-fixed, paraffin embedded (FFPE) archive material. Successful miRNA-seq of RNA extracted from FFPE material has previously been reported and we therefore proceeded with the library preparation despite the high degree of RNA degradation [33–35]. The stability of small RNAs such as miRNAs and piRNAs did allow us to successfully prepare the small RNA libraries with a clear enrichment of fragments with the expected size of approx. 145 bp (Supplementary Figure 1B). The small RNA libraries were subsequently sequenced and the average distribution of detected RNA species is shown in Figure 1. Degradation of larger RNA species, such as ribosomal RNA (rRNA) and messenger RNAs (mRNAs), did influence the output from the small RNA sequencing as 32.7% and 12.5% of sequence reads were determined to originate from rRNA and mRNA, respectively. However, 14.4% and 10.9% of sequencing reads were still detected from miRNAs and piRNAs, respectively.

### Characterization of the miRNA transcriptome in metastasizing and non-metastasizing LAC

A full list of all miRNAs detected by miRNA-seq sorted according to total mean expression in all samples is provided in Supplementary Table 2. A total of 189 miRNAs demonstrated a mean expression level of more than 100 counts per million (CPM) miRNA reads and the expression levels of these miRNAs were compared between the LACs without distant metastases and LACs with distant metastases. The miRNAs were sorted according to the most significant change in expression between the two groups and the results are shown in Supplementary Table 3 and visualized as a heatmap in Supplementary Figure 2. Due to the size similarity between miRNA and piRNA, we also obtained full expression profile for piRNA. A complete list of all detected piRNA sorted according to total mean expression in all samples is shown in Supplementary Table 4. 120 piRNAs showed a mean expression level of more than 100 counts per million piRNA counts (CPM) and their expression was compared between the LACs with and without distant metastases. The piRNAs were then sorted by the most significant change in expression between the two groups and the results are shown in Supplementary Table 5 and depicted as a heatmap in Supplementary Figure 3.

**Figure 2:**
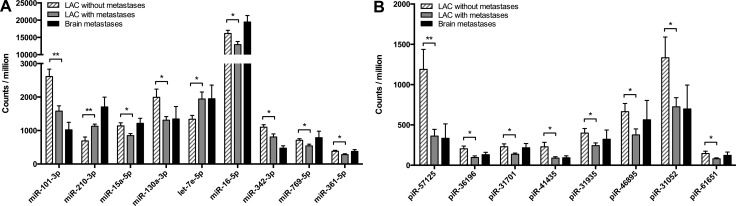
Differentially expressed miRNAs and piRNAs determined by miRNA-seq The miRNA and piRNA expression levels determined by miRNA-seq were compared between the primary tumor groups “LAC w/ metastases” and “LAC w/o metastases” using a student's *t*-test with two-tailed *p*-values ≤ 0.05 considered statistically significant. The miRNAs and piRNAs that exhibited a significant change in expression between the two primary tumor groups are shown in (**A**) for miRNAs and in (**B**) for piRNAs. The results are shown as bar graphs representing the mean expression in each groups (counts per million miRNA/piRNA counts) with error bars indicating the standard error of the mean (SEM).

Only subtle differences in expression were detected between the metastasizing and non-metastasizing LACs for both miRNAs and piRNA (Supplementary Figures 2 and 3). Nine miRNAs demonstrated a significant change in expression between the two groups: miR-101-3p (*p* = 0.0020), miR-210-3p (*p* = 0.0051), miR-15a-5p (*p* = 0.0195), miR-130a-3p (*p* = 0.0227), let-7e-5p (*p* = 0.0235), miR-16-5p (*p* = 0.0246), miR-342-3p (*p* = 0.0262), miR-769-5p (*p* = 0.0272) and miR-361-5p (*p* = 0.0273) (Figure 2A). Similarly, a significant change in expression was detected for 8 piRNA: piR-57125 (*p* = 0.0068), piR-36196 (*p* = 0.0172), piR-31701 (*p* = 0.0313), piR-41435 (*p* = 0.0325), piR-31935 (*p* = 0.0326), piR-46895 (*p* = 0.0396), piR-31052 (*p* = 0.0477) and piR-61651 (*p* = 0.0494) (Figure 2B). Similar changes in expression were observed in the paired brain metastases for the majority of the miRNAs and piRNA, but due to the considerable difference in average tumor cell content between the primary tumors and brain metastases, the expression results could not be compared directly (Supplementary Table 1). Based upon the miRNA-seq analysis, we selected 10 miRNAs that showed a significant or near significant difference in expression between the metastasizing and non-metastasizing LACs to undergo validation by RT-qPCR. The 10 selected miRNAs, including mean CPM, standard error of the mean (SEM) and *p*-value determining the significance of the difference in expression are shown in Table 1.

**Table 1:** miRNA-seq results for miRNAs selected for RT-qPCR validation

ID	LAC w/o metastases		LAC w/ metastases		Brain metastases		LAC w/o metastases vs. LAC w/ metastases
	Mean (CPM)	SEM	Mean (CPM)	SEM	Mean (CPM)	SEM	*P*
miR-101-3p	2612	221	1579	159	1021	223	**0.0020**
miR-210-3p	689	116	1128	63	1704	294	**0.0051**
miR-15a-5p	1138	91	847	62	1214	151	**0.0195**
miR-130a-3p	1992	245	1309	107	1345	372	**0.0227**
miR-16-5p	16161	913	12909	914	19478	1874	**0.0246**
miR-342-3p	1100	75	806	92	472	71	**0.0262**
miR-361-5p	380	29	279	29	377	53	**0.0273**
miR-30a-3p	1245	298	576	120	371	68	0.0559
miR-10b-5p	10195	1826	24564	6965	15307	4573	0.0658
miR-100-5p	11291	3983	3342	262	3090	907	0.0664

### High concordance between miRNA-Seq and RT-qPCR in FFPE material

**Figure 3:**
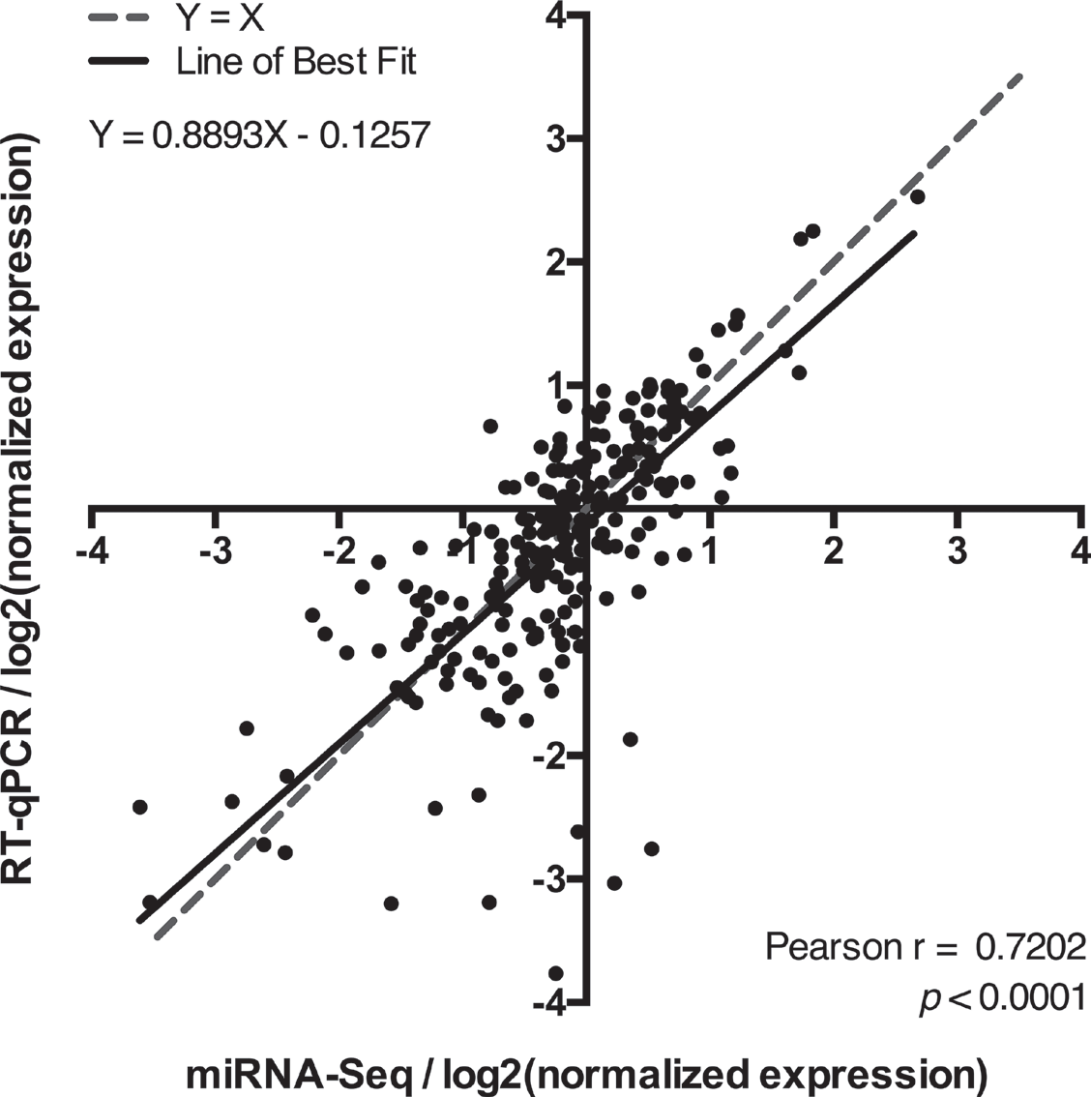
The level of agreement between miRNA-seq and RT-qPCR The level of agreement between the miRNA-seq and RT-qPCR expression results was assessed by plotting the log2(normalized expression) values obtained by RT-qPCR for each miRNA vs. those obtained by miRNA-seq for all of the 24 patient samples that were included in the miRNA-seq analysis. The level of agreement between the two methodologies was determined by linear regression and correlation analysis. The resulting equation for the Line of Best Fit, as well as the Pearson's correlation coefficient, r, and corresponding *p*-value are shown.

This study was conducted using highly degraded RNA extracted from FFPE archive material (Supplementary Figure 1), and we therefore performed an initial RT-qPCR validation to confirm our miRNA-seq results. The expression level of the 10 selected miRNAs was determined by RT-qPCR in the same 24 patient samples and the miRNA-seq and RT-qPCR expression values were then normalized to enable direct comparison. For each miRNA in each of the 24 samples, log2 converted normalized expression values were generated for the two methods separately: For RT-qPCR, normalized expression values were generated by dividing the RQ of a given miRNA in each sample to the mean RQ of said miRNA in all 24 samples and subsequently log2 converted. For miRNA-seq, normalized expression values were generated by dividing the CPM of a given miRNA in each sample to the mean CPM of said miRNA in all 24 samples and subsequently log2 converted. The log2 (normalized expression) values obtained by RT-qPCR were then plotted against those obtained by miRNA-seq (Figure 3). The level of agreement between the two methodologies was determined by correlation analysis and linear regression. The correlation analysis yielded a Pearson correlation coefficient, *r* = 0.7202 and a *p*-value < 0.0001 thus showing a strong correlation between the results obtained by the two methods. Similarly, the linear regression determined the Line of Best Fit as Y = 0.8893X – 0.1257, which approximates the ideal line, Y = X. We can therefore conclude that there is a high concordance between the results obtained by miRNA-seq and RT-qPCR despite the high level of RNA degradation present in FFPE material.

### Validation of differentially expressed miRNAs

**Figure 4:**
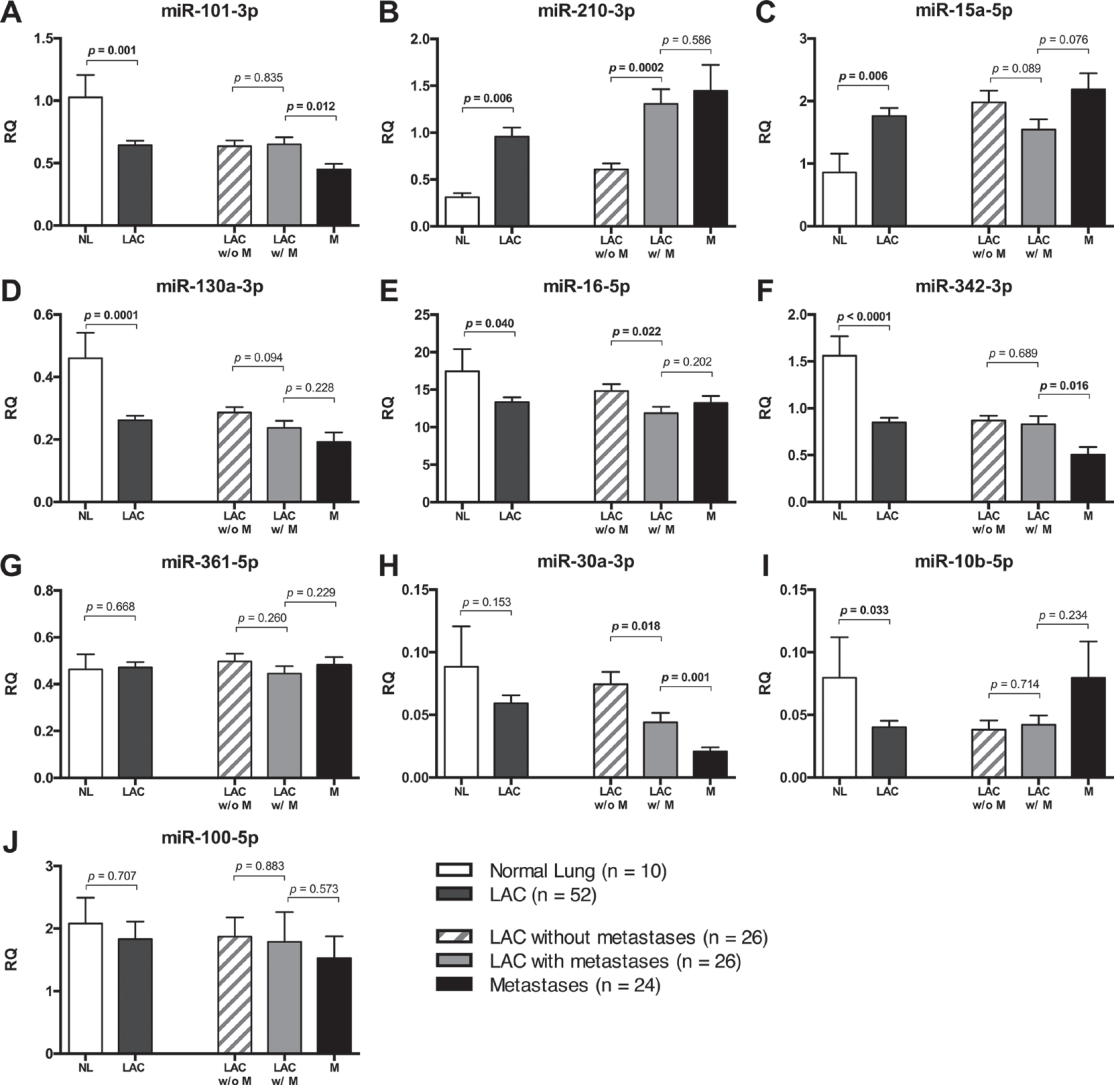
Validation of differentially expressed miRNAs The relative quantities (RQ) of the 10 miRNAs that were selected for validation were determined in 26 non-metastasizing (LAC w/o M) and 26 metastasizing LACs (LAC w/ M), as well as in 24 paired distant metastases (20 brain and 4 adrenal gland) (M), and 10 tumor-adjacent normal lungs (NL) using RT-qPCR. The “LAC” group represents the mean expression level in the non-metastasizing and metastasizing LACs combined. The results are shown as bar graphs representing the mean expression level in each group with error bars indicating the standard errors of the mean (SEM) for (**A**) hsa-miR-101-3p, (**B**) hsa-miR-210-3p, (**C**) hsa-miR-15a-5p, (**D**) hsa-miR-130a-3p, (**E**) hsa-miR-16-5p, (**F**) hsa-miR-342-3p, (**G**) hsa-miR-361-5p, (**H**) hsa-miR-30a-3p, (**I**) hsa-miR-10b-5p and in (**J**) for hsa-miR-100-5p. The statistical significance was determined using unpaired student's *t*-tests with two-tailed *p*-values ≤ 0.05 considered statistically significant.

Following the initial validation, we then determined the expression of the 10 selected miRNAs by RT-qPCR in the full LAC cohorts comprising LACs from 26 distant metastases-free patients and LACs from 26 patients diagnosed with distant metastases, as well as in 24 paired metastases (20 brain and 4 adrenal gland) and 10 tumor-adjacent normal lung samples. The clinical characteristics of the patients included in the study are listed in Supplementary Table 6. All miRNAs selected for validation, except miR-361-5p and miR-100-5p, showed a significant change in expression between tumor-adjacent normal lung and LAC. Two miRNAs (miR-210-3p and miR-15a-5p) were significantly upregulated and 6 miRNAs (miR-101-3p, miR-130a-3p, miR-16-5p, miR-342-3p, miR-30a-3p and miR-10b-5p) were downregulated in the tumor tissue compared to the normal counterpart (Table 2 and Figure 4). A statistically significant change in expression between metastasizing and non-metastasizing LACs was confirmed for three miRNAs; miR-210-3p, miR-30a-3 and miR-16-5p, with similar or more pronounced changes in expression levels observed in the paired distant metastases. Next, we analyzed publicly available microRNA expression datasets through The Cancer Genome Atlas (TGCA) database (https://gdc-portal.nci.nih.gov/). Expression values were collected for all miRNAs that demonstrated a significant change in expression between tumor-adjacent normal lung and tumor tissue, or between metastasizing and non-metastasizing LACs in our LAC cohorts. We first analyzed the expression levels in 46 LAC patients with paired tumor-adjacent normal lung and primary tumor samples available, validating that miR-210-3p is significantly upregulated, whereas miR-101-3p and miR-30a-3p are significantly downregulated in LAC compared to normal lung (Supplementary Figure 4). No difference in expression was detected for miR-15a-5p, miR-130a-3p, miR-16-5p, miR-342-3p and miR-10b-5p in the TCGA samples. Next, we compared the expression levels of the three miRNAs that exhibited differential expression in our metastasizing and non-metastasizing LACs in the TCGA samples with and without distant metastases (M1, *n* = 23 vs. M0, *n* = 346), TCGA samples with and without lymph node metastases (N1 or N2, *n* = 123 vs. N0, *n* = 219) and TCGA samples from patients with and without recurrent disease (YES, *n* = 123 vs. NO, *n* = 219). For miR-210-3p, we did not find a significant association between the expression levels and the included clinical parameters, but a clear tendency towards higher expression in the metastasizing vs. non-metastasizing LACs was observed (mean miRNA expression: M1 = 1629 ± 385 CPM (*n* = 23) vs. M0 = 1231 ± 68.68 CPM (*n* = 346)) (Supplementary Figure 5A). For miR-30a-3p, we detected a borderline significant association between low expression levels and the presence of lymph node metastases (*p* = 0.062) and disease recurrence (*p* = 0.065) (Supplementary Figure 5B). No association between miR-16-5p expression levels and the included clinical parameters was observed in the TCGA samples (Supplementary Figure 5C). In combination, these results strongly suggest that upregulation of miR-210-3p and downregulation of miR-30a plays an important role in the development and metastatic progression of LAC.

**Table 2:** miRNAs demonstrating differential expression

ID	LAC vs. Normal lung		LAC w/ metastases vs.LAC w/o metastases	
	Change in expression	*P*	Change in expression	*P*
miR-101-3p	Down	0.001	-	-
miR-210-3p	Up	0.006	Up	0.0002
miR-15a-5p	Up	0.006	-	-
miR-130a-3p	Down	0.0001	-	-
miR-16-5p	Down	0.040	Down	0.022
miR-342-3p	Down	< 0.0001	-	-
miR-361-5p	-	-	-	-
miR-30a-3p	Down	0.153	Down	0.018
miR-10b-5p	Down	0.033	-	-
miR-100-5p	-	-	-	-

### Increased miR-210 expression is associated with the presence of distant metastases in LAC

**Figure 5:**
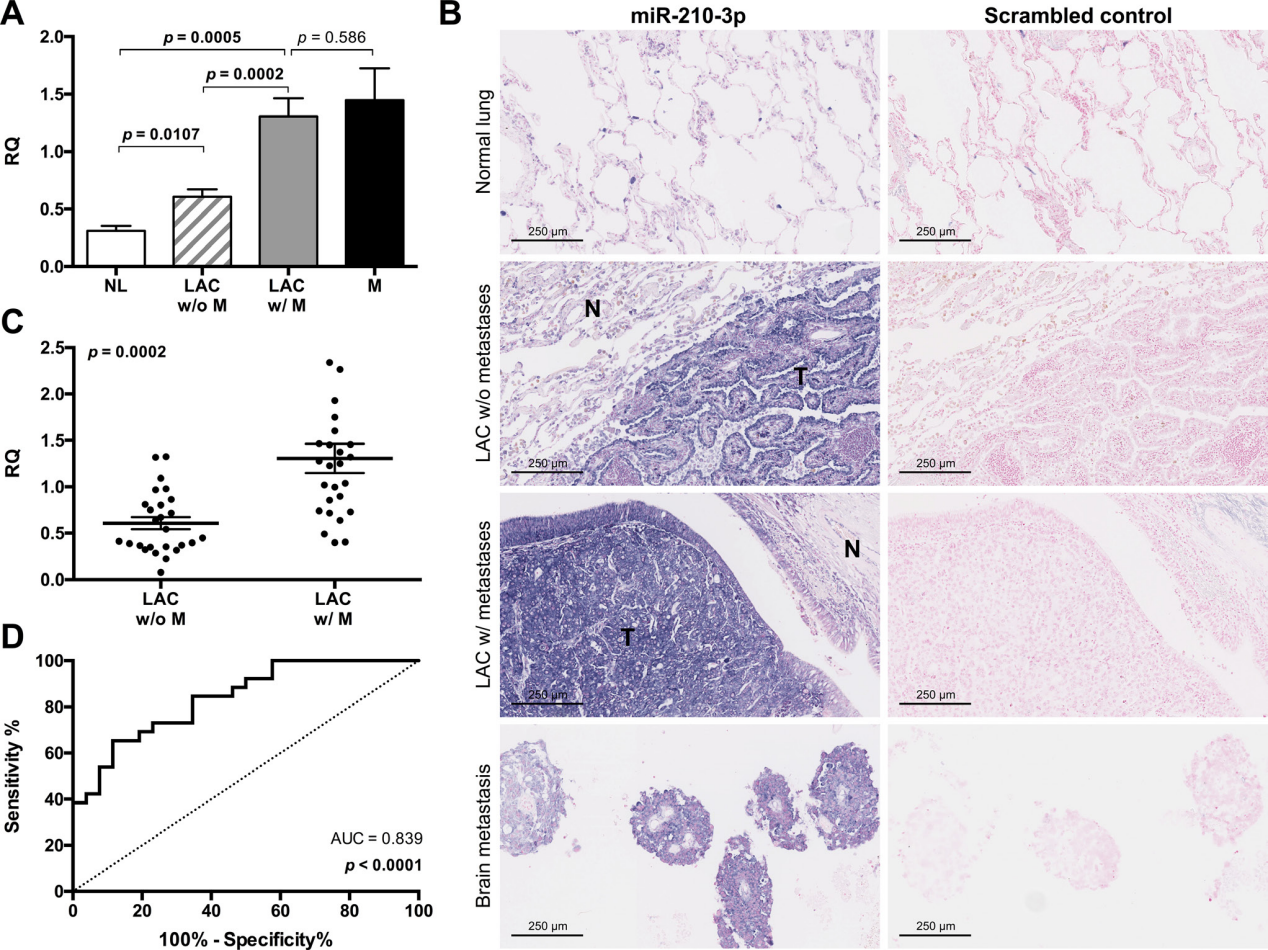
Increased expression of miR-210 is associated with distant metastasis in LAC The association between miR-210-3p expression and distant metastasis in LAC was investigated. (**A**) The relative quantity (RQ) of miR-210-3p was determined by RT-qPCR and shown as a bar graph representing the mean expression in each group with error bars indicating the standard error of the mean (SEM). (**B**) The expression of miR-210-3p was investigated by microRNA *in situ* hybridization in 3 tumor-adjacent normal lung samples, 3 LACs without metastases, 3 LAC with distant metastases and 3 paired brain metastasis. A scrambled probe was included as a negative control. Representative images from each sample group are shown. (**C**) The RQ of miR-210-3p determined by RT-qPCR in the metastasizing (LAC w/ M) and non-metastasizing (LAC w/o M) LAC are shown as a dot plot for visualization of variation in expression within each group. Unpaired student's *t*-tests were used to compare miR-210-3p expression between groups and two-tailed *p*-values ≤ 0.05 considered statistically significant. (**D**) A Receiver Operating Characteristic (ROC) curve was used to determine the potential of miR-210-3p expression as a biomarker for distant metastasis in LAC. The Area Under the Curve (AUC) was calculated and the resulting *p*-value (Null hypothesis: AUC = 0.5) is indicated.

miR-210-3p demonstrated the most significant change in expression between metastasizing and non-metastasizing LACs and we therefore chose this miRNA for further characterization. miR-210-3p was significantly upregulated in both the non-metastasizing (*p* = 0.0107) and metastasizing LACs (*p* = 0.0005) as compared to the tumor adjacent normal lungs (Figure 5A). The expression of miR-210-3p was furthermore significantly higher in the metastasizing tumors compared to the non-metastasizing tumors (*p* = 0.0002) and similarly increased levels of expression were detected in the paired distant metastases. The RNA used in this study was extracted from whole sections of FFPE surgical resections, which contain contaminating normal lung cells (Average proportion of tumor cells in primary LACs = 33.2%). In order to determine if the increased expression of miR-210-3p was specific for the tumor cells in the sections, we investigated miR-210-3p expression by *in situ* hybridization in 3 tumor-adjacent normal lung samples, 3 LACs without metastases, 3 LAC with metastases and in 3 paired brain metastases. The areas of high miR-210-3p expression (dark purple) co-localize clearly with the tumor tissue (Figure 5B). We can therefore conclude that the observed increase in miR-210 expression is specific for the tumor cells in the sections. The correlation between miR-210-3p expression and the clinical characteristics of the patients included in this study, such as gender, age, smoking status, tumor size (T-stage), presence of regional lymph node metastases (N-stage) and presence of distant metastases (M-Stage) was investigated (Table 3). M-stage (*p* = 0.0002) was the only clinical parameter to show a significant correlation and increased miR-210-3p expression is thus specifically associated to the presence of distant metastases in LAC. The variation in miR-210-3p expression in the metastasizing and non-metastasizing LACs is shown in Figure 5C. In order to determine if miR-210-3p expression holds potential as a diagnostic biomarker for distant metastasis in LAC, we performed a Receiver Operating Characteristic (ROC) curve analysis. It showed an area under the ROC curve (AUC) of 0.839 (*p* < 0.0001), which confirmed the high potential of miR-210-3p expression as a biomarker for formation of distant metastases in LAC (Figure 5D).

**Table 3:** miR-210-3p expression vs. clinicopathological parameters

Characteristic		miR-210-3p expression		
	N	Mean	SEM	*P*
**Gender**				
Male	22	1.036	0.190	0.4908
Female	30	0.898	0.099	
**Age**				
< Average (61.7 years)	25	0.863	0.112	0.3594
> Average (61.7 years)	27	1.044	0.157	
**Smoking status**				
Current Smoker	34	0.970	0.137	0.5704
Previous Smoker	16	0.847	0.117	
**T-Stage (TNM)**				
T1	18	0.844	0.127	0.4486
T2–T4	33	1.003	0.137	
**N Stage (TNM)**				
N0	33	0.991	0.143	0.8503
N1–N2	17	0.950	0.107	
**M-Stage (TNM)**				
M0	26	0.608	0.066	**0.0002**
M1	26	1.306	0.158	

### Increased miR-210 expression as a consequence of promoter hypomethylation

**Figure 6:**
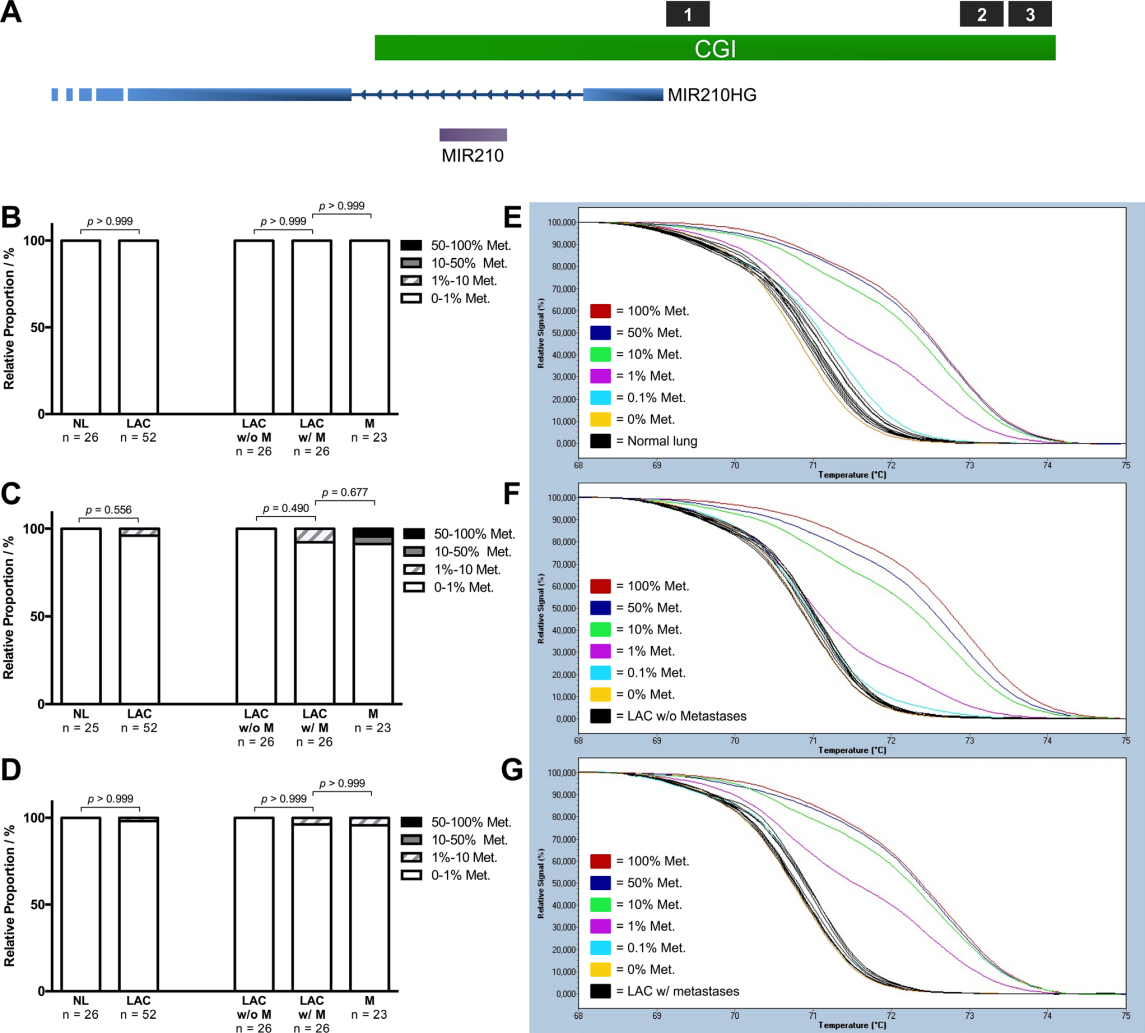
Methylation assessment of the miR-210 promoter region The methylation status of Region 1, 2 and 3 of the miR-210-3p promoter region was assessed in 26 metastasizing (LAC w/ M) and 26 non-metastasizing lung adenocarcinomas (LAC w/o M), as well as in 26 tumor-adjacent normal lung samples (NL) using MS-HRM analysis. “LAC” denotes the metastasizing and non-metastasizing LACs combined. A schematic representation of the genomic context of miR-210 is shown in (**A**) with the location of Region 1, 2 and 3 within the CpG Island (CGI) indicated in black. The results of the methylation assessment are visualized as stacked bar percentage plots in (**B**) for Region 1, in (**C**) for Region 2 and in (**D**) for Region 3. In each category, the relative proportion of samples with 0-1% methylated templates are indicated in white, 1–10% methylated templates in white with light grey stripes, 10–50% methylated templates in dark grey and samples with 50–100% methylated templates are indicated in black. For Region 1, normalized melting profiles for the DNA methylation standards and (**E**) 10 tumor-adjacent normal lung samples, (**F**) 10 non-metastasizing LACs and (**G**) 10 metastasizing LACs are shown in representative singlicates. The 100% methylated standard is indicated in red, 50% methylated standard in dark blue, 10% methylated standard in green, 1% methylated standard in purple, 0.1% methylated standard in light blue and the 0 % methylated standard in yellow. The statistical significance was assessed using a Mann-Whitney test of ranks with two-tailed *p*-values ≤ 0.05 considered significant.

The *miR-210* gene is located in an intron of the miR-210 host gene (*MIR210HG*), which coincides with a CpG island (CGI). Hypoxia-induced increased expression of miR-210 has previously been attributed to promoter hypomethylation, and we therefore hypothesized that the increased expression observed in the metastasizing tumors could be a consequence of a decrease in promoter methylation [36]. We therefore investigated the methylation status of three different regions of the CGI using Methylation-Sensitive High Resolution Melting (MS-HRM) analysis. A schematic representation of the miR-210's genomic context is shown in Figure 6A with the location of the three MS-HRM assays targeting Region 1, 2 and 3, indicated in black. The three regions were chosen based on their likelihood of being of transcriptional relevance for miR-210/MIR210HG when inspecting of the UCSC Genome Browser (Human Feb. 2009 GRCh37/hg19 Assembly). Regions with a high density of transcription factor binding sites, high DNAase hypersensitivity and H3K27Ac marks were prioritized [37]. The results of the methylation assessment are shown in Supplementary Table 7 and visualized as stacked bar percentage plots in Figure 6B for Region 1, Figure 6C for Region 2 and in Figure 6D for Region 3. Representative normalized melting profiles are shown for Region 1 in Figure 6E–6G. No significant difference in methylation was detected between any of the sample groups and largely all samples were unmethylated at all three regions. For Region 1, 100% (101/101) of the tested samples were unmethylated. For Region 2, 100% (25/25) of the tumor-adjacent normal lung samples and 100% (26/26) LACs without metastases were unmethylated, whereas a non-significant increase in methylation was detected in 7.7% (2/26) of the LACs with metastases and in 8.8% (2/23) of the distant metastases. Similar results were obtained for Region 3, as 100% (26/26) of the tumor-adjacent normal lung samples and 100% (26/26) of the LACs without metastases were unmethylated, whereas 3.9% (1/26) of the LACs with metastases and 4.4% (1/23) of the distant metastases showed an increase in methylation. We can therefore conclude that the observed increase in miR-210 expression is not a consequence of aberrant DNA methylation at any of the three tested regions.

## DISCUSSION

Lung cancer is responsible for the most cancer-related deaths worldwide and the majority of these deaths are caused by metastatic disease. Identification of the molecular mechanisms leading to lung cancer metastasis may therefore hold the key to lowering the mortality of the disease. The non-coding RNA transcriptome is an area of increasing scientific interest and its critical role in human carcinogenesis is continuously being uncovered [38, 39]. This study is, to the best of our knowledge, the first to interrogate the entire miRNA transcriptome in relation to lung cancer metastasis by miRNA-seq and provides a full profile of all miRNAs that are expressed in metastasizing and non-metastasizing lung adenocarcinomas, as well as information regarding their relative abundance in these tissues. We compared the miRNA transcriptomes of the metastasizing and non-metastasizing LACs and while only subtle differences in miRNA expression were detected, we identified nine miRNAs that demonstrated a significant change in expression between the two groups: miR-101-3p, miR-210-3p, miR-15a-5p, miR-130a-3p, let-7e-5p, miR-16-5p, miR-342-3p, miR-769-5p and miR-361-5p.

Due to the size similarity between miRNA and piRNA, we also obtained full profiles of the piRNA transcriptomes in metastasizing and non-metastasizing lung adenocarcinomas, which provide important information concerning the role and abundance of piRNA in human malignancies. Eight piRNAs exhibited differential expression between the metastasizing and non-metastasizing LACs. The most pronounced difference was detected for piR-57125, which was significantly downregulated in the metastasizing LACs as compared to the non-metastasizing LACs (*p* = 0.0068). Interestingly, piR-57125 was also recently shown to be significantly downregulated in metastasizing compared to non-metastasizing clear cell renal cell carcinomas [40]. These results strongly suggest that piR-57125 may function as a suppressor of cancer metastasis and it would therefore be highly interesting to functionally characterize this small RNA, as well as to investigate if this is a common observation in other metastasizing solid tumors.

In the present study, 10 miRNAs demonstrating a significant or near-significant difference in expression between the metastasizing and non-metastasizing tumors were selected to undergo validation in our LAC cohorts by RT-qPCR. We were able to confirm a statistically significant difference in expression between metastasizing and non-metastasizing LACs for miR-30a-3p, miR-16-5p and miR-210-3p with similar or more pronounced changes in expression levels observed in the paired distant metastases. Both miR-30a-3p and miR-16-5p were significantly downregulated in the metastasizing tumors, which suggest that they could function as tumor suppressors and inhibit metastasis in LAC. miR-210-3p exhibited the strongest association with the presence of distant metastases in LAC patients. We found that miR-210-3p was upregulated in both the metastasizing and non-metastasizing LACs as compared to tumor-adjacent normal lung, with its expression being significantly higher in the metastasizing than in the non-metastasizing LACs. To further ensure the validity of our findings, we analyzed the publicly available LAC miRNA expression datasets in the TGCA database, which confirmed that miR-210-3p and miR-30a-3p are indeed significantly up- and downregulated, respectively, in LAC compared to paired tumor-adjacent normal lung samples. While we did not detect a statistically significant association between the expression of miR-30a-3p, miR-16-5p and miR-210-3p and distant metastatic disease in the TCGA samples, we did find a borderline significant association between low miR-30a-3p expression levels and the presence of lymph node metastases and disease recurrence, which are traits of aggressive tumor behavior. We moreover found that the mean expression level of miR-210-3p was more than 1.3 fold higher in the metastasizing vs. non-metastasizing LACs, and we therefore believe that the lack of significant association is most likely due to the rather small sample size of the TCGA M1 group (M1, *n* = 23 vs. M0 = 346). Combined, these results strongly indicate that upregulation of miR-210-3p and downregulation of miR-30a-3p play important roles in the development and metastatic progression of LAC.

Downregulation of miR-30a is, in fact, a common observation in various malignancies, including lung cancer, and it has been shown to inhibit epithelial-to-mesenchymal transition (EMT) and metastasis, as well as to be associated with poor prognosis in NSCLC [41–44]. miR-210 is a direct transcriptional target of HIF-1a and is induced in response to hypoxia [45]. Upregulation of miR-210 is thus a frequent observation in cancer and increased expression has been identified as an independent negative prognostic factor in several cancers, including breast, head-and-neck, pancreas, and lung cancer [19, 20, 46–50]. In LAC, increased expression of miR-210 in primary tumors has been shown to be significantly associated with a high risk of relapse and increased serum levels have been found associated with the presence of regional lymph node metastases in NSCLC [50, 51]. Increased expression of miR-210 has also been linked to metastatic progression in colon, stomach and liver cancer, and elevated plasma levels has been identified as a potential biomarker for early detection of metastatic disease in melanoma and breast cancer [52–56]. This study is, to the best of our knowledge, the first to link increased expression of miR-210-3p to the development of distant metastases in LAC and it therefore adds valuable information to the increasing amount of evidence of miR-210s critical role in cancer development and metastatic progression.

Patient specimens are routinely formalin-fixed and paraffin embedded (FFPE) in pathology laboratories across the world, but the fixation procedure induces extensive DNA and RNA degradation, which limits its use for downstream molecular analyses. However, the vast abundance of archived FFPE patient material renders it a valuable type of research material and it is therefore equally important to recognize its potential as its limitations. Previous studies have reported successful miRNA-seq of FFPE material and our study serves to confirm this notion. Despite the high level of RNA degradation observed in our samples, we still obtained valuable data that demonstrated a high level of concordance with results obtained by RT-qPCR [33]. We therefore wish to highlight the potential of FFPE material to study genome-wide changes in miRNA expression by miRNA-seq.

In conclusion, this study provides a full profile of the miRNA and piRNA transcriptome in metastasizing and non-metastasizing LAC, thus bringing novel insight to the role of ncRNA in the pathogenesis of lung cancer. We have shown that upregulation of miR-210-3p and downregulation of miR-30a-3p are significantly associated with the formation of distant metastases and these changes therefore represent attractive candidates for novel biomarkers of distant metastasis in LAC. It would be highly relevant to validate our findings in a large retrospective cohort study, as well as to investigate the predictive value of these miRNAs in prospective cohort studies. Moreover, it would be interesting for future studies to investigate if these changes can be detected in non-invasive patient samples, such as blood, expectorates or bronchial washings, as this could potentially allow them to be applied as diagnostic biomarkers for detection of undiscovered micrometastases, as well as for monitoring metastases-related recurrence in surgically resected patients. Lastly, it would be relevant to functionally characterize these miRNAs in relation to cancer metastasis, as well as to determine their potential as targets for novel miRNA inhibition or replacement therapies for prevention of metastases formation in LAC.

## MATERIALS AND METHODS

### Patient samples

Formalin-fixed paraffin embedded (FFPE) surgical resections from 52 lung adenocarcinoma (LAC) patients were retrieved from the archives at the Department of Pathology, Aarhus University Hospital. The patients were divided into two cohorts depending on the existence of distant metastatic disease at the time of diagnosis. The first cohort comprised primary lung adenocarcinomas from 26 patients without distant metastatic disease and a minimum of 5 years of recurrence-free survival following surgery. The second cohort comprised primary lung adenocarcinomas and paired distant metastases (20 brain and 4 adrenal gland) from 26 patients with distant metastatic disease. The cohorts were matched on histology, tumor size (T-stage), gender, age, smoking status and the relative proportion of tumor cells in the surgical resections. Tumor-adjacent normal lung tissue from 26 patients was included as controls. The Regional Ethical Committee (De Videnskabsetiske Komitéer Region Midtjylland, Permission No.: 1-10-72-20-14) approved this study and all data was analyzed anonymously in accordance with the approval.

### RNA and DNA extraction

From each FFPE sample, 1 × 7 μm and 5 × 10 μm sections were cut for RNA and DNA extraction, respectively. RNA was extracted using the miRNeasy FFPE kit (Qiagen, Hilden, Germany) and eluted in a final volume of 17 μL RNase-free water according to the manufacturer´s instructions. Potential genomic DNA contamination in the eluted RNA was removed by DNase treatment. DNA was extracted using the QIAamp DNA FFPE Tissue Kit (Qiagen, Hilden, Germany) according to the manufacturer's protocol. RNA and DNA concentrations including OD (A260/280) were assessed using the NanoDrop 1000 (Thermo Scientific, Waltham, MA, USA).

### Small RNA library preparation and sequencing

The RNA integrity in each sample was evaluated using the Agilent Bioanalyzer RNA 6000 Nano assay (Agilent Technologies, Santa Clara, CA, USA). Small RNA libraries were constructed using TruSeq Small RNA Sample Prep Kit (Illumina, San Diego, CA, USA) following the manufacturer´s instructions. From each sample, 500 ng RNA was ligated to 3′ and 5′ adapters and then subjected to reverse transcription to obtain cDNA. The cDNA was amplified for 12 PCR cycles using a common primer and a primer containing an index tag. The amplified libraries were subsequently gel purified. The size and purity of the obtained libraries were validated using the Agilent Bioanalyzer High Sensitivity DNA assay (Agilent Technologies, Santa Clara, CA, USA) and the concentrations were confirmed using the KAPA Library Quantification Kit (KAPA Biosystems, Wilmington, MA, USA). The libraries were subsequently pooled and sequenced on an Illumina Hiseq2000 instrument by the Beijing Genomics Institute (BGI).

### miRNA-Seq analysis

Raw sequencing reads were filtered with FASTX-Toolkit to trim away low-quality reads and cutadapt to remove adaptor sequences. The clean reads were mapped to a list of datasets using Bowtie. First, reads were mapped to miRNAs from miRBase v21 allowing zero mismatches, disregarding non-templated A/U tailing on mature miRNAs. Non-miRNA-mapping reads were subsequently matched against other relevant small RNA datasets: piRNA, tRNA, snRNA, snoRNA and Y RNA allowing one mismatch. To assess degradation, the remaining unmatched reads were mapped to long RNA datasets: rRNA, other RNAs from Rfam and refSeq mRNA. The expressions of miRNAs and piRNAs were normalized as counts per million miRNA/piRNA reads (CPM). Only miRNAs/piRNAs expressed above 100 CPM were used for further analysis. Heatmaps were performed in R using the Bioconductor package ComplexHeatmap. All miRNA/piRNA with mean expressions above 100 CPM were visualized in ascending order by *p*-value between the groups “without metastases” and “with metastases”. Data was log2 transformed and rows were mean centered prior to visualization.

### cDNA synthesis and RT-qPCR

cDNA synthesis and RT-qPCR were performed using the miRCURY LNA^TM^ Universal RT microRNA PCR system (Exiqon, Vedbaek, Denmark) according to the manufacturer's instructions. The cDNA synthesis was performed in duplicates for each sample with 10 ng RNA as input. The RT-qPCR was conducted on a LightCycler® 480 (Roche, Mannheim, Germany) in a final volume of 5 μL comprising 2 × ExiLENT SYBR^®^ master mix, cDNA and LNA™ PCR primers for hsa-miR-101-3p, hsa-miR-210-3p, hsa-miR-15a-5p, hsa-miR-130a-3p, hsa-miR-16-5p, hsa-miR-342-3p, hsa-miR-361-5p, hsa-miR-30a-3p, hsa-miR-10b-5p, hsa-miR-100-5p and two miRNA reference genes, hsa-miR-103a-3p and hsa-miR-423-5p [57, 58]. All expression data was analyzed using NormFinder to ensure the stability of the reference genes [59]. ΔCt values were calculated by normalizing to the geometric mean of the reference genes. Relative quantities (RQ) were calculated as 2^−ΔCt^ and the RQ of the miRNAs of interest in each sample was determined as the mean RQ in the cDNA synthesis duplicates.

### miRNA expression analysis using the cancer genome atlas (TCGA) database

Clinical information and miRNA expression data for hsa-miR-101-1 (3p), hsa-miR-101-2 (3p), hsa-miR-210-3p, hsa-miR-15a-5p, hsa-miR-130a-3p, hsa-miR-16-1 (5p), hsa-miR-16-2 (5p), hsa-miR-342-3p, hsa-miR-30a-3p, hsa-miR-10b-5p were retrieved from https://gdc-portal.nci.nih.gov/ for more than 400 lung adenocarcinoma patients.

### miR *in situ* hybridization

miR-210-3p expression was analyzed by *in situ* hybridization using a 5′- and 3′-double digoxigenin-labeled miRCURY LNA^TM^ microRNA Detection Probe (Exiqon, Vedbaek, Denmark) with minor modifications to the manufacturer's protocol. U6 and a scrambled probe were included as positive and negative controls, respectively. In brief, the deparaffinized and proteinase K-digested sections were dehydrated and hybridized with 40 nM double-DIG LNA^TM^ hsa-miR-210-3p probe for 60 min at 54°C. Following a series of stringent saline-sodium citrate buffer washes and blocking, the sections were incubated with a 1:125 dilution of anti-DIG Fab fragments conjugated to alkaline phosphatase (Roche Diagnostics, Mannheim, Germany). The anti-DIG/AP was replaced with fresh reagent after 30 min and the incubation was continued for an additional 30 min. For signal detection, the sections were incubated with freshly prepared NBT/BCIP AP substrate (Roche Diagnostics, Mannheim, Germany) at 30°C. After 60 min, fresh AP substrate was added and the incubation was continued for 60 min. Lastly, Nuclear Fast Red^TM^ (Vector laboratories, Burlingame, CA, USA) was used as a counterstain for visualization of the nuclei.

### Sodium bisulfite modification and methylation-sensitive high resolution melting (MS-HRM) analysis

From each sample, 500 ng genomic DNA was subjected to sodium bisulfite modification using the EZ DNA Methylation-Gold™ Kit (Zymo Research, Irvine, CA, USA) and eluted in a final volume of 110 μl H_2_O according to the manufacturer's instructions. Methylation assessment of Region 1, 2 and 3 of the miR-210 promoter was performed by MS-HRM analysis and the primers were designed to amplify both methylated and unmethylated DNA [60–62]. PCR and HRM were carried out on a LightCycler^®^ 480 (Roche, Mannheim, Germany) with each 10 μl reaction comprising 2 × MeltDoctor^TM^ HRM Master Mix (Life Technologies, Carlsbad, CA, USA), 3 mM MgCl_2_, approximately 10 ng bisulfite modified DNA and 500 nM of each primer. The methylation assessment of each region was performed by melting profile comparison between each sample and DNA methylation standards derived through a serial dilution of 100% methylated DNA (Universal Methylated Human DNA Standard, Zymo Research, Irvine, CA USA) into 0% methylated DNA (EpiTect Control DNA, Qiagen, Hilden, Germany). All analyses were done in triplicates and the technical specifications for the Region 1, 2 and 3 MS-HRM assays, including primer sequences, genomic location, assay-specific PCR cycling and HRM protocols are listed in Supplementary Methods.

### Statistical analysis

The miRNA expression values determined by miRNA-seq and RT-qPCR were compared between groups using unpaired Student's *t*-tests. The level of agreement between miRNA-seq and RT-qPCR was determined through linear regression and correlation analysis of the log2(normalized expression) values obtained by RT-qPCR plotted against the log2(normalized expression) values obtained by miRNA-seq. To obtain the log2(normalized expression) values, the expression value for each miRNA was normalized to the mean miRNA expression in all 24 samples used for miRNA-seq and the values were then log2 transformed. Receiver Operating Characteristic curve analysis was used to determine the biomarker potential of miR-210-3p expression. miRNA expression data obtained from the TCGA database was compared between samples with and without distant metastases, with and without lymph node metastases and samples from patients with and without recurrent disease using a Mann-Whitney test of ranks, and between paired tumor-adjacent normal lung and tumor tissue samples using a Wilcoxon matched pairs signed rank test. The methylation frequencies of Region 1, 2 and 3 were compared between groups using a Mann-Whitney test of ranks. Samples were ranked according to their determined level of methylation with samples demonstrating 0–1% methylation ranked 1, 1–10% methylation ranked 2, 10–50% methylation ranked 3 and 50–100% methylation ranked 4. All analyses were conducted using GraphPad Prism version 6 software (GraphPad Software, La Jolla, CA, USA) with two-tailed *p*-values ≤ 0.05 considered statistically significant.

## SUPPLEMENTARY MATERIALS FIGURES AND TABLES










