# Supplementary material for: Small RNA sequencing reveals metastasis-related microRNAs in lung adenocarcinoma
Source: Oncotarget. 2017 Mar 7;8(16):27047–61. doi: 10.18632/oncotarget.15968 (PMC5432317; doi:10.18632/oncotarget.15968)
Supplement: Supplementary file 1 [file oncotarget-08-27047-s001.pdf]

Small RNA sequencing reveals metastasis-related microRNAs in lung adenocarcinoma

Supplementary Materials

SUPPLEMENTARY METHODS

MS-HRM Assays

The technical specifications, including primer sequences, amplicon length, genomic location (UCSC Genome Browser on Human Feb. 2009 (GRCh37/hg19) Assembly) and assay-specific PCR and HRM protocols are shown below for the three MS-HRM assays covering Region 1, 2 and 3 of the miR-210 promoter. For each MS-HRM assay, 180 bp of surrounding genomic sequence (upper strand) and corresponding bisulfite-modified sequence (lower strand) is shown with the locations of the primers indicated in red. The transcription start site for the miR-210 host gene (MIR210HG) is indicated in yellow.

Normalized melting profiles for the DNA methylation standards are shown in representative technical duplicates for each MS-HRM assay. The DNA methylation standards were generated through a serial dilution of 100% methylated DNA into 0% methylated DNA. The 100% methylated standard is indicated in red, 50% methylated standard in dark blue, 10% methylated standard in green, 1% methylated standard in pink, 0.1% methylated standard in light blue and the 0% methylated standard in orange.

Region 1

PCR and HRM protocol for the Region 1 MS-HRM assay: 1 cycle of 95ºC for 10 minutes. 1 cycle of 95ºC for 15 seconds, 45 cycles of 1 minute at 57ºC. 95°C for 1 minute, 55ºC for 1 minute and a melting phase from 55°C to 95°C with a temperature increase of 0.01°C/sec and 50 fluorescence acquisition points per °C. Amplicon length = 81 bp.

Genomic location (Hg19): Chr11: 568421-568600

```
GCCCCCCGGCCCGACCCGACCGGCCCCGGGCCCCGCGGCTCCACGCCCAGCCCCGTATGC
|:::::++|::++|::++|:++|:::++||:::++++|:|::|++:::||:::++||||:
GTTTTTCGGTTCGATTCGATCGGTTTCGGGTTTCGCGGTTTTACGTTTAGTTTCGTATGT

AAATGACCTGGTCCCTCAGCCAATGGCCGGCCTAGCCGGGCCCGCCCTACCGCAACGCAG
||||||::||||:::|:||::|||||:++|::|||:++||::++:::||:++:||++:||
AAATGATTTGGTTTTTTAGTTAATGGTCGGTTTAGTCGGGTTCGTTTTATCGTAACGTAG

CCAGTGGGCGCGAGGGGGGTTCGCGACGCCCCGCCCACCGCCGCGCGGCCCTTCGCGGGC
::||||||++++|||||||||++++|++:::++:::|:++:++++++|:::||++++||:
TTAGTGGGCGCGAGGGGGGTTCGCGACGTTTCGTTTATCGTCGCGCGGTTTTTCGCGGGT
```

MS-HRM Primers:

Region 1 F: 5′ – GTTTCGTATGTAAATGATTTGG – 3′

Region 1 R: 5′ – CGCCCACTAACTACATTAC – 3′

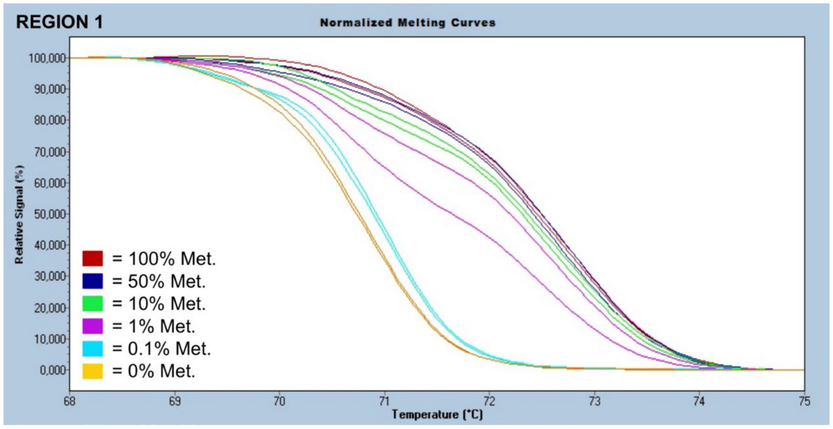

Region 2

PCR and HRM protocol for the Region 2 MS-HRM assay: 1 cycle of 95ºC for 10 minutes. 1 cycle of 95ºC for 15 seconds, 45 cycles of 1 minute at 60ºC. 95°C for 1 minute, 55ºC for 1 minute and a melting phase from 55°C to 95°C with a temperature increase of 0.01°C/sec and 50 fluorescence acquisition points per °C. Amplicon length = 134 bp.

Genomic location (Hg19): Chr11: 569171-569350

```
GCTCTGTCTCCGCGGAAGCCTCTGGAGCCTGGAGGGGGTGCCTCAAGGGCTCAGGGGTCT
|:|:|||:|:++++||||::|:|||||::|||||||||||::|:|||||:|:||||||:|
GTTTTGTTTTCGCGGAAGTTTTTGGAGTTTGGAGGGGGTGTTTTAAGGGTTTAGGGGTTT

TCGCAGCCCTGTGCCGGTCTTTGAGGGGACGAGGCGAGAGAATCCTGTCTGTGCGCTGAA
|++:||:::||||:++||:||||||||||++|||++|||||||::|||:||||++:||||
TCGTAGTTTTGTGTCGGTTTTTGAGGGGACGAGGCGAGAGAATTTTGTTTGTGCGTTGAA

AACGTGTAAAATCAAAGGAGCCTTGACGGTTTGACCTTCGTTAGGACTCGCAGGGCCAGC
||++||||||||:|||||||::||||++||||||::||++||||||:|++:||||::||:
AACGTGTAAAATTAAAGGAGTTTTGACGGTTTGATTTTCGTTAGGATTCGTAGGGTTAGT
```

MS-HRM Primers:
Region 2 F: 5′ – CGGAAGTTTTTGGAGTTTGG – 3′
Region 2 R: 5′ – TCAAAACTCCTTTAATTTTACAC – 3′

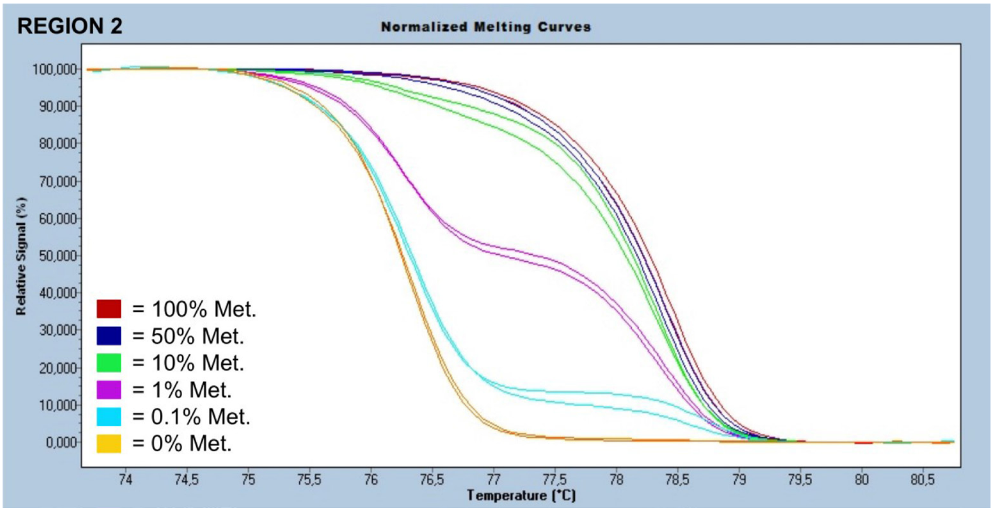

Region 3

PCR and HRM protocol for the Region 3 MS-HRM assay: 1 cycle of 95ºC for 10 minutes. 1 cycle of 95ºC for 15 seconds, 45 cycles of 1 minute at 58ºC. 95°C for 1 minute, 55ºC for 1 minute and a melting phase from 55°C to 95°C with a temperature increase of 0.01°C/sec and 50 fluorescence acquisition points per °C. Amplicon length = 120 bp.

Genomic location (Hg19): Chr11: 569278-569457

```
CCGGGGCCGAGGACCAGGGTGACAGTGCCAAGGGGGGCCGGCGGCCAAATCTCCTTTGGC
:++|||:++||||::|||||||:||||::||||||||:++|++|::||||:|::|||||:
TCGGGGTCGAGGATTAGGGTGATAGTGTTAAGGGGGGTCGGCGGTTAAATTTTTTTTGGT

TGCCACTTTGGAGCCCCACCCCGGAGTCCGATACTTCCACGTCTGAAGCTGGCCCTGCGA
||::|:|||||||::::|:::++||||:++|||:||::|++|:|||||:|||:::||++|
TGTTATTTTGGAGTTTTATTTCGGAGTTCGATATTTTTACGTTTGAAGTTGGTTTTGCGA

GTCCTAACGAAGGTCAAACCGTCAAGGCTCCTTTGATTTTACACGTTTTCAGCGCACAGA
||::|||++|||||:|||:++|:||||:|::||||||||||:|++||||:||++:|:|||
GTTTTAACGAAGGTTAAATCGTTAAGGTTTTTTTGATTTTATACGTTTTTAGCGTATAGA
```

MS-HRM Primers:

Region 3 F: 5′ – GTCGAGGATTAGGGTGATAG – 3′

Region 3 R: 5′ – AAAACTCGCAAAACCAACTTC – 3′

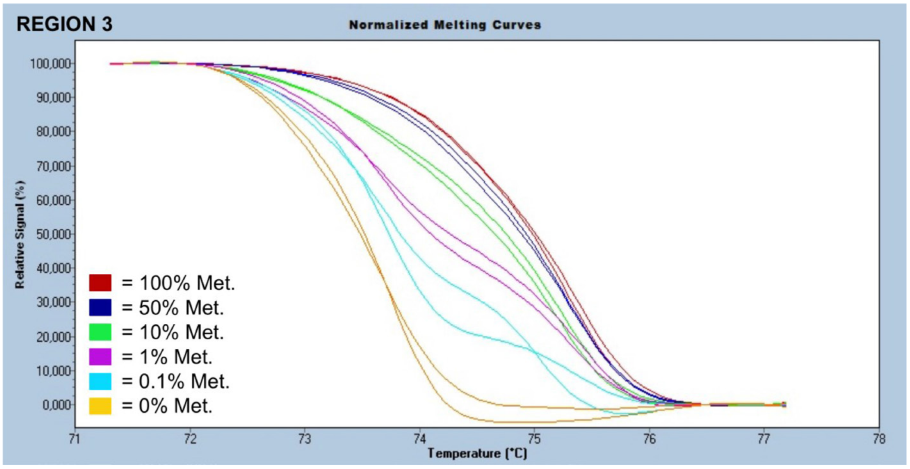

LAC w/o metastases (Samples 1A-8A)
LAC w/ metastases (Samples 1B-8B)
Metastases (Samples 1C-8C)

A Assesment of RNA integrity

Agilent Bioanalyzer: RNA 6000 Nano assay

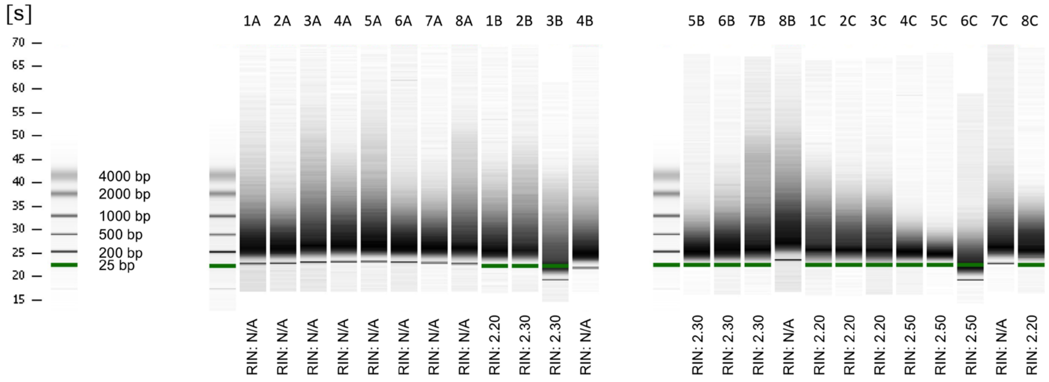

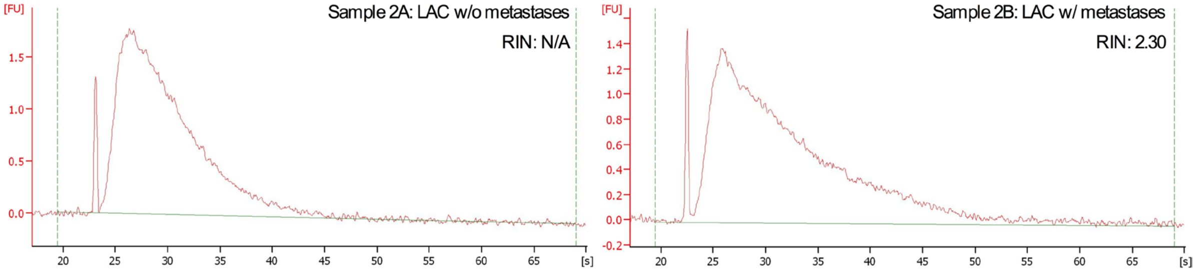

B Assesment of library size and purity

Agilent Bioanalyzer: High Sensitivity DNA Assay

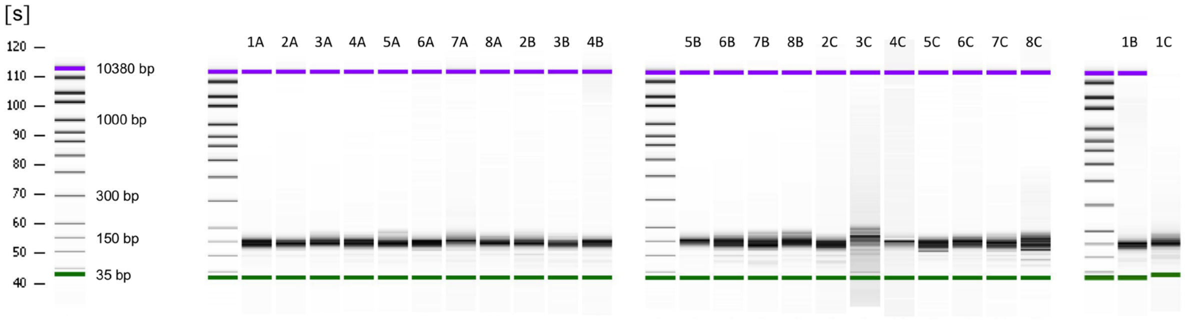

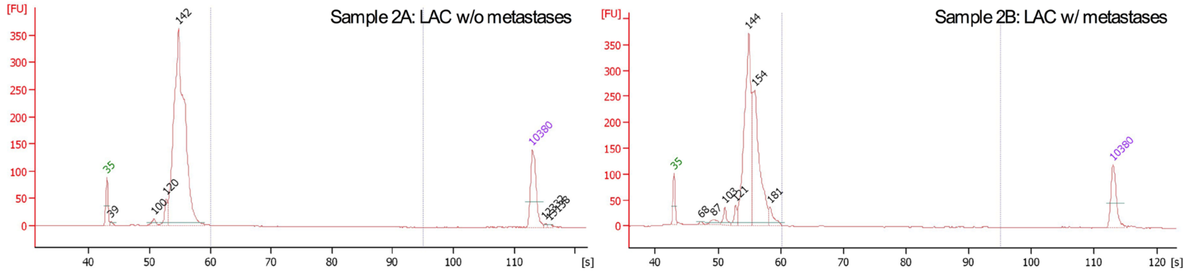

Supplementary Figure 1: RNA integrity and small RNA library validation. The RNA integrity of the 24 patient samples used for miSeq was assessed on an Agilent Bioanalyzer and the results are shown in (**A**). As it can be observed, all samples demonstrated a high degree of RNA degradation. Similarly, the size and purity of the generated small RNA libraries were validated on an Agilent Bioanalyzer as shown in (**B**).

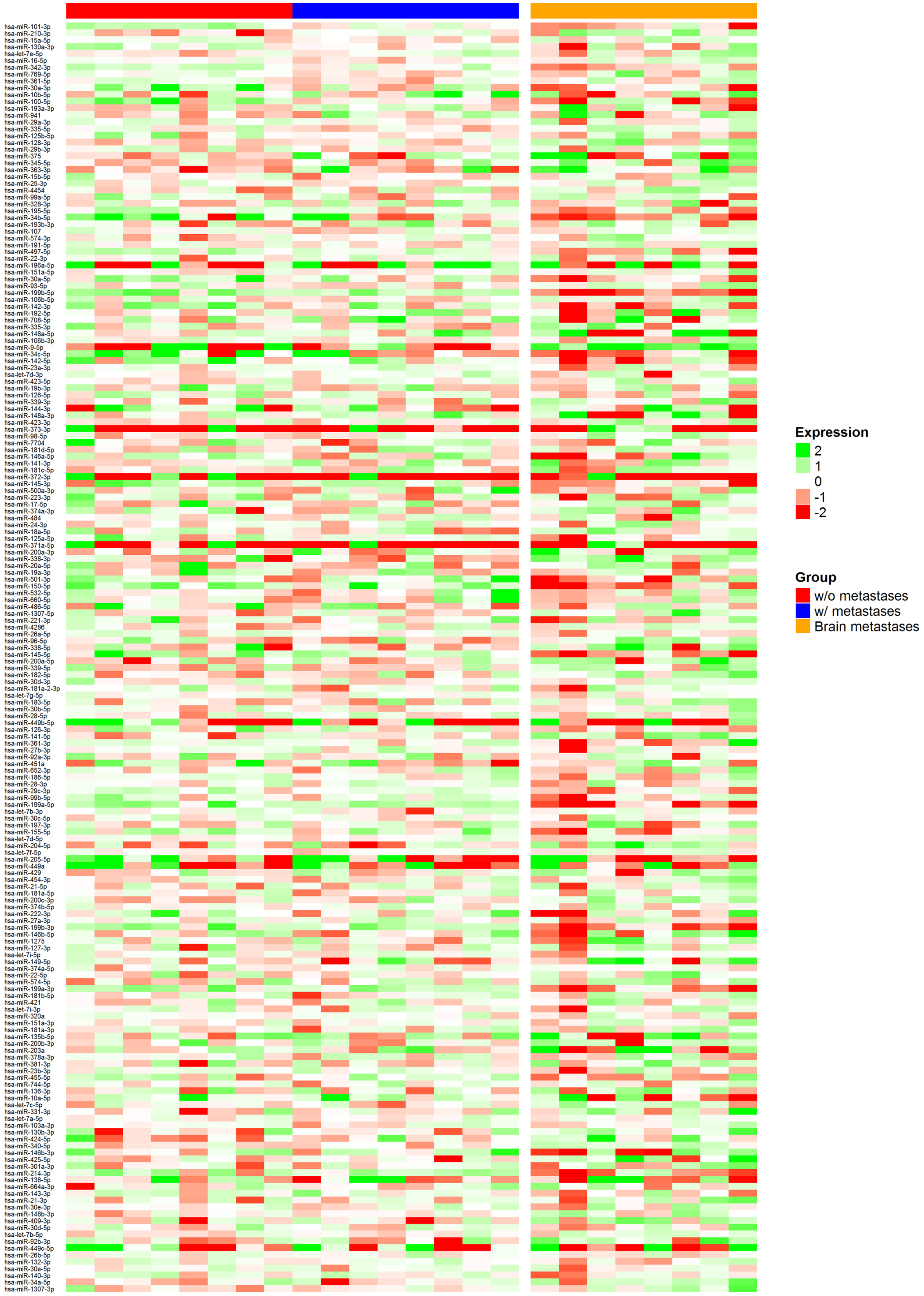

Supplementary Figure 2: miRNA expression profile in metastasizing and non-metastasizing LAC. The miRNA transcriptomes in 8 LACs from patients without distant metastases (LAC w/o metastases), 8 LACs from patients with distant metastases (LAC w/ metastases) and 8 paired brain metastases were characterized using miRNA-seq. The expression of all miRNAs with a total mean expression level (all groups combined) of ≥ 100 count per million miRNA counts was compared between the tumor groups “LAC w/ metastases” and “LAC w/o metastases” using a student’s *t*-test. The miRNAs were sorted by *p*-value (low to high) and the results are depicted as a heat map with green indicating samples with increased expression and red samples with decreased miRNA expression.

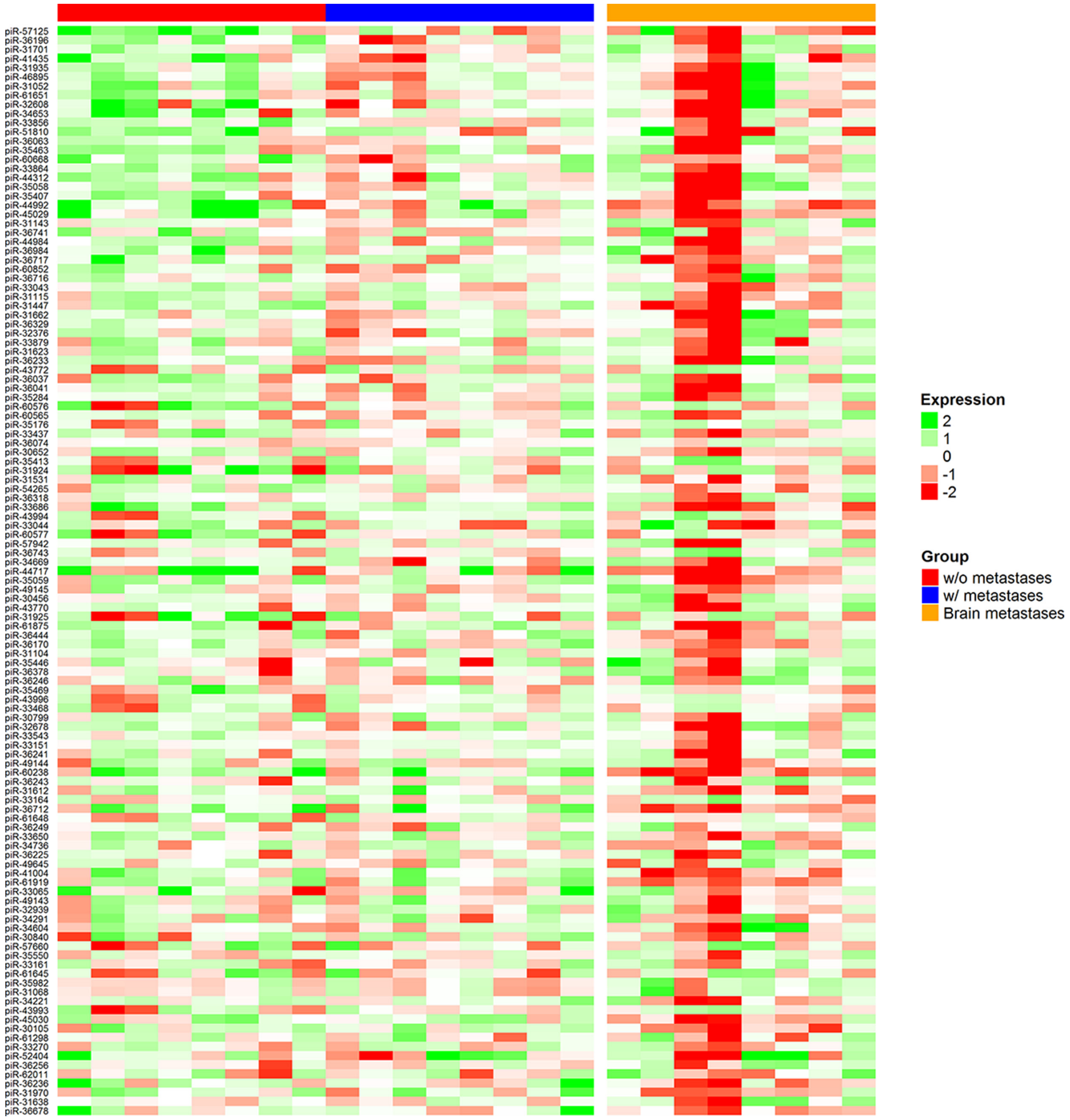

Supplementary Figure 3: piRNA expression profile in metastasizing and non-metastasizing LAC. The piRNA transcriptomes in 8 LACs from patients without distant metastases (LAC w/o metastases), 8 LACs from patients with distant metastases (LAC w/ metastases) and 8 paired brain metastases were characterized using miRNA-seq. The expression of all piRNAs with a total mean expression level (all groups combined) of ≥ 100 count per million piRNA counts was compared between the tumor groups "LAC w/ metastases" and "LAC w/o metastases" using a student's *t*-test. The piRNAs were sorted by *p*-value (low to high) and the results are depicted as a heat map with green indicating samples with increased expression and red samples with decreased piRNA expression.

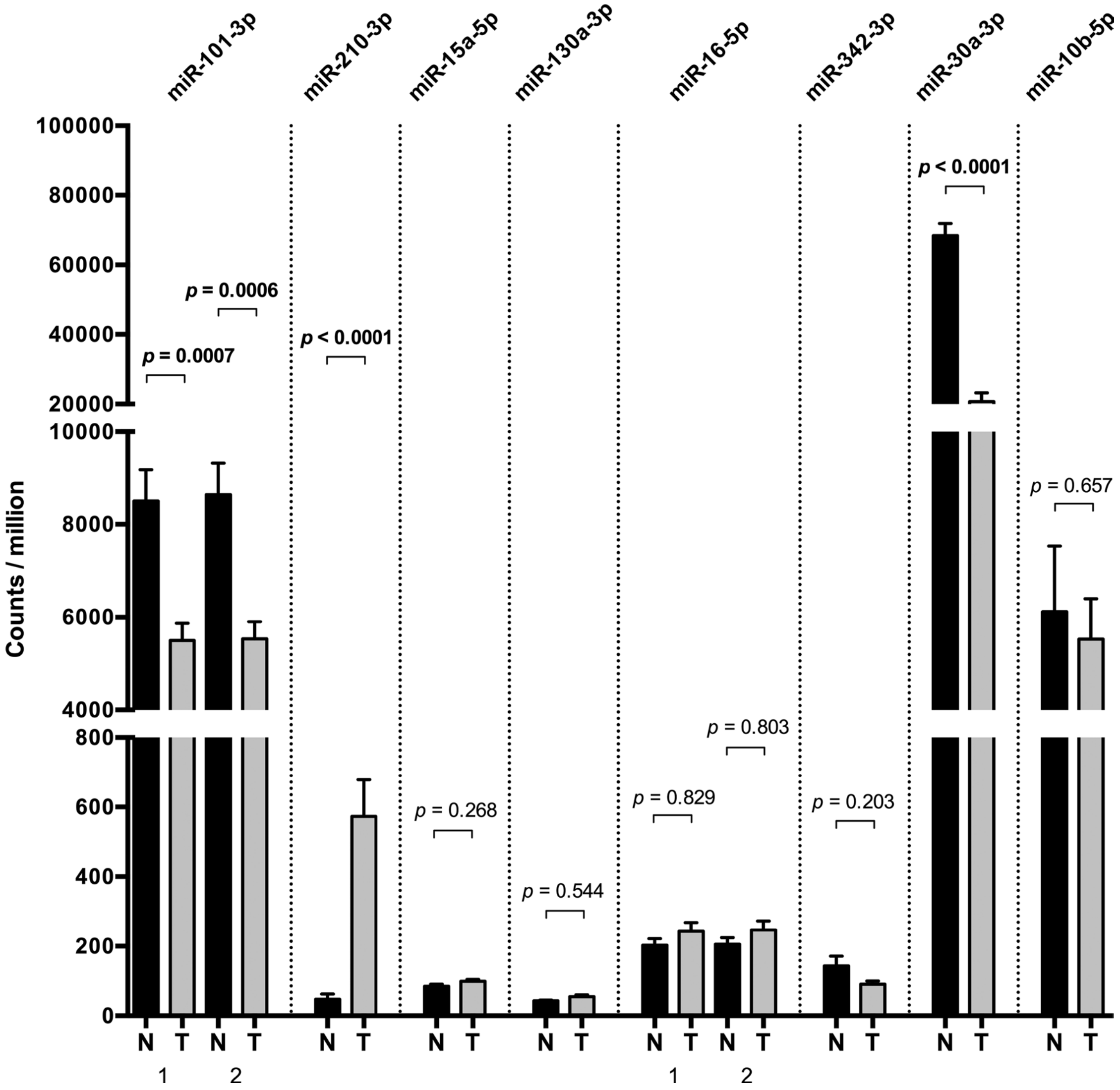

Supplementary Figure 4: miRNA expression in paired LAC and tumor-adjacent normal lung samples obtained from the TCGA database. miRNA expression data for hsa-miR-101-3p (hsa-miR-101-1 (1) and hsa-miR-101-2 (2)), hsa-miR-210-3p, hsa-miR-15a-5p, hsa-miR-130a-3p, hsa-miR-16-5p (hsa-miR-16-1 (1) and hsa-miR-16-2 (2)), hsa-miR-342-3p, hsa-miR-30a-3p, hsa-miR-10b-5p were retrieved from https://gdc-portal.nci.nih.gov/ for 46 LAC patients with paired tumor (T) and tumor-adjacent normal lung (N) samples available. For each miRNA, expression levels were compared between groups using a Wilcoxon matched pairs signed rank test.

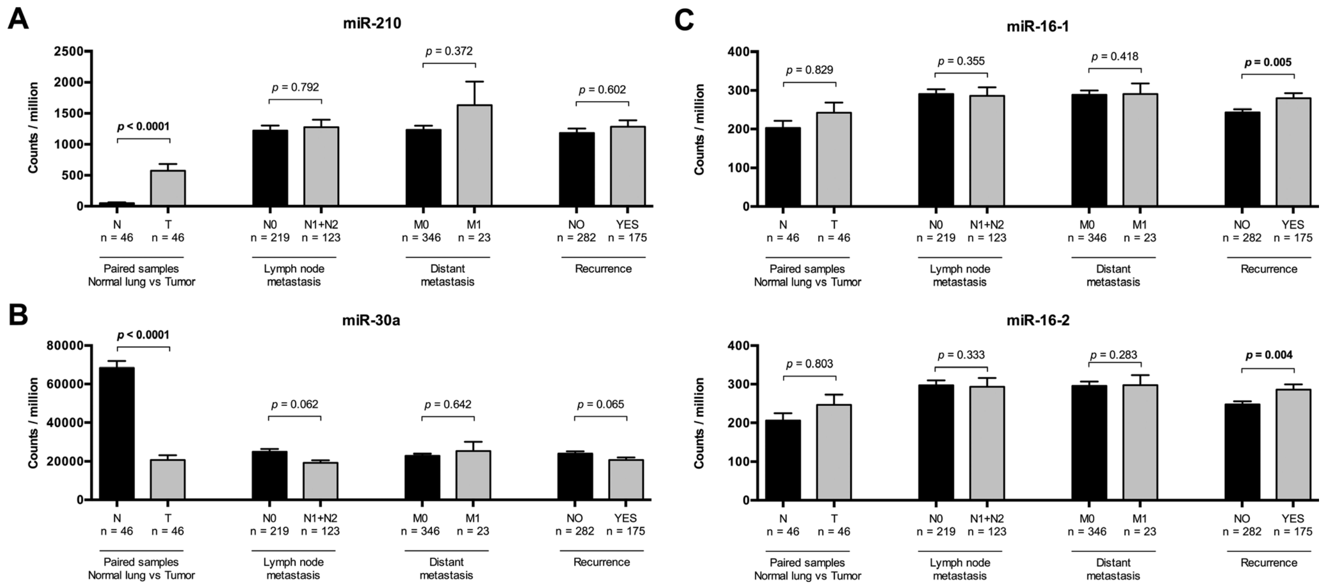

Supplementary Figure 5: miRNA expression in 400+ LAC samples obtained from the TCGA database. Clinical information and miRNA expression data for (**A**) hsa-miR-210-3p, (**B**) hsa-miR-30a-3p and (**C**) hsa-miR-16-5p (upper panel: hsa-miR-16-1, lower panel: hsa-miR-16-2) were retrieved from https://gdc-portal.nci.nih.gov/ for more than 400 lung adenocarcinoma patients. miRNA expression levels were compared between paired tumor (T) and tumor-adjacent normal lung (N) samples using Wilcoxon matched pairs signed rank test, as well as between samples with and without lymph node metastases (N1 or N2, n = 123 vs. N0, n = 219), samples with and without distant metastases (M1, n = 23 vs. M0, n = 346) and samples from patients with and without recurrent disease (YES, n = 123 vs. NO, n = 219) using a Mann-Whitney test of ranks.

Supplementary Table 1: Clinical characteristics of the patients included in miRNA-Seq

Clinical Characteristics	LAC w/o metastases	LAC w/ metastases
**Patients (*n*)**		
Cases	8	8
Cases with paired distant metastases	0	8
**Gender (*n* (%))**		
Male	4 (50.0%)	4 (50.0%)
Female	4 (50.0%)	4 (50.0%)
**Age (years)**		
Min-Max (Average)	45–75 (61.1)	42–70 (60.3)
**TNM Classification (*n* (%))**		
T1	4 (50.0%)	4 (50.0%)
T2	4 (50.0%)	4 (50.0%)
T3	0 (0%)	0 (0%)
T4	0 (0%)	0 (0%)
T Unknown	0 (0%)	0 (0%)
N0	8 (100%)	2 (25.0%)
N1	0 (0%)	2 (25.0%)
N2	0 (0%)	4 (50.0%)
N3	0 (0%)	0 (0%)
N Unknown	0 (0%)	0 (0%)
M0	8 (100%)	0 (0%)
M1	0 (0%)	8 (100%)
**Smoking status (*n* (%))**		
Current Smoker	5 (62.5%)	5 (62.5%)
Previous Smoker	3 (37.5%)	3 (37.5%)
Unknown	0 (0%)	0 (0%)
**Tumor content (%)**		
Tumors, Min-Max (Average)	20–40% (31.3%)	30–60% (41.3%)
Metastases, Min-Max (Average)		50–80% (70.0%)

Supplementary Table 2: miRNA expression in LAC. See Supplementary_Table_2

Supplementary Table 3: miRNA expression in LAC sorted by *p*-value. See Supplementary_Table_3

Supplementary Table 4: piRNA expression in LAC. See Supplementary_Table_4

Supplementary Table 5: piRNA expression in LAC sorted by *p*-value. See Supplementary_Table_5

Supplementary Table 6: Clinical characteristics for the LAC cohorts

Clinical Characteristics	LAC w/o metastases	LAC w/ metastases
**Patients (*n*)**		
Cases	26	26
Cases with paired distant metastases	0	24
**Gender (*n* (%))**		
Male	10 (38.5%)	12 (46.2%)
Female	16 (61.5%)	14 (53.8%)
**Age (years)**		
Min-Max (Average)	45–76 (62.1)	38–76 (61.2)
**TNM Classification (*n* (%))**		
T1	9 (34.7%)	9 ((34.7%)
T2	16 (61.5%)	16 (61.5%)
T3	0 (0%)	0 (0%)
T4	1 (3.8%)	0 (0%)
T Unknown	0 (0%)	1 (3.8%)
N0	21 (80.8%)	12 (46.2%)
N1	5 (19.2)	3 (11.4%)
N2	0 (0%)	9 (34.7%)
N3	0 (0%)	0 (0%)
N Unknown	0 (0%)	2 (7.7%)
M0	26 (100%)	0 (0%)
M1	0 (0%)	26 (100%)
**Smoking status (*n* (%))**		
Current Smoker	18 (69.2%)	16 (61.5%)
Previous Smoker	8 (30.8%)	8 (30.8%)
Unknown	0 (0%)	2 (7.7%)
**Tumor content (%)**		
Tumors, Min-Max (Average)	5–60% (27.0%)	5–80% (39.4%)
Metastases, Min-Max (Average)		5–90% (68.7%)

Supplementary Table 7: Methylation assessment of region 1, 2 and 3

Sample Type	Methylation level	Region 1<br>*n* (%)	Region 2<br>*n* (%)	Region 3<br>*n* (%)
**Normal lung**	0–1%	26/26 (100%)	25/25 (100%)	26/26 (100%)
	1–10%	–	–	–
	10–50%	–	–	–
	50–100%	–	–	–
**LAC**	0–1%	52/52 (100%)	50/52 (96.2%)	51/52 (98.1%)
	1–10%	–	2/52 (3.8%)	1/52 (1.9%)
	10–50%	–	–	–
	50–100%	–	–	–
**LAC w/o Metastases**	0–1%	26/26 (100%)	26/26 (100%)	26/26 (100%)
	1–10%	–	–	–
	10–50%	–	–	–
	50–100%	–	–	–
**LAC w/ Metastases**	0–1%	26/26 (100%)	24/26 (92.3%)	25/26 (96.1%)
	1–10%	–	2/26 (7.7%)	1/26 (3.9%)
	10–50%	–	–	–
	50–100%	–	–	–
**Metastases**	0–1%	23/23 (100%)	21/23 (91.2%)	22/23 (95.6%)
	1–10%	–	–	1/23 (4.4%)
	10–50%	–	1/23 (4.4%)	–
	50–100%	–	1/23 (4.4%)	–
